# General practitioners’ experiences and perceptions of benzodiazepine prescribing: systematic review and meta-synthesis

**DOI:** 10.1186/1471-2296-14-191

**Published:** 2013-12-13

**Authors:** Coral Sirdifield, Sibyl Anthierens, Hanne Creupelandt, Susan Y Chipchase, Thierry Christiaens, Aloysius Niroshan Siriwardena

**Affiliations:** 1 Community and Health Research Unit, School of Health and Social Care, University of Lincoln, Lincoln, UK; 2 Department of Primary Health Care and Interdisciplinary Care, University of Antwerp, Antwerp, Belgium; 3 Department of General Practice and Primary Health Care, Ghent University, Ghent, Belgium; 4 School of Psychology, University of Lincoln, Lincoln LN6 7TS, UK; 5 Department of Family Medicine and Primary Health Care and Heymans Institute of Pharmacology, Ghent University, Ghent, Belgium; 6 Community and Health Research Unit, School of Health and Social Care, University of Lincoln, Lincoln, UK

**Keywords:** Sleep initiation and maintenance disorders, Review, Benzodiazepines, Primary health care, Therapeutics, Inappropriate prescribing

## Abstract

**Background:**

Benzodiazepines are often prescribed long-term inappropriately. We aimed to systematically review and meta-synthesise qualitative studies exploring clinicians’ experiences and perceptions of benzodiazepine prescribing to build an explanatory model of processes underlying current prescribing practices.

**Methods:**

We searched seven electronic databases for qualitative studies in Western primary care settings published in a European language between January 1990 and August 2011 analysing GP or practice nurse experiences of benzodiazepine prescribing. We assessed study quality using the Critical Appraisal Skills Programme Checklist. We analysed findings using thematic synthesis.

**Results:**

We included eight studies from seven countries published between 1993 and 2010. Benzodiazepine prescribing decisions are complex, uncomfortable, and demanding, taken within the constraints of daily general practice. Different GPs varied in the extent to which they were willing to prescribe benzodiazepines, and individual GPs’ approaches also varied. GPs were ambivalent in their attitude towards prescribing benzodiazepines and inconsistently applied management strategies for their use. This was due to the changing context of prescribing, differing perceptions of the role and responsibility of the GP, variation in GPs’ attitudes to benzodiazepines, perceived lack of alternative treatment options, GPs’ perception of patient expectations and the doctor-patient relationship. GPs faced different challenges in managing initiation, continuation and withdrawal of benzodiazepines.

**Conclusion:**

We have developed a model which could be used to inform future interventions to improve adherence to benzodiazepine prescribing guidance and improve prescribing through education and training of professionals on benzodiazepine use and withdrawal, greater provision of alternatives to drugs, reflective practice, and better communication with patients.

## Background

Benzodiazepines are used to treat conditions such as insomnia, anxiety and chronic back pain. They have considerable adverse effects including memory disruption, increased risk of accidents and falls, and dependence [1,2]. Despite guidance advocating use of psychological treatments first-line before drug treatment for insomnia [3], and short-term use of drugs when these are used [4,5], numerous studies have shown that benzodiazepines are being overprescribed for extended periods [6-8] in many countries. For example, a review encompassing studies of benzodiazepine use in primary care in European and other countries concluded that “the use of benzodiazepines in the long-term is a very common phenomenon” [9]. Many have questioned why this is the case, and what influences clinicians’ decisions whether or not to initiate, continue or withdraw a benzodiazepine prescription.

Benzodiazepine prescribing rates vary between clinicians and practices [10], partly explained by patient demographics, clinician attributes and differences in general practice organisation [10-15]. Previous research has also demonstrated that clinicians’ attitudes and experiences affect their adherence to clinical guidelines [16].

We aimed to explore clinicians’ experiences and perceptions of primary care benzodiazepine prescribing, and to build an explanatory model of the processes underlying clinicians’ benzodiazepine prescribing using a meta-synthesis of qualitative studies.

## Methods

We conducted a systematic review of qualitative studies exploring clinicians’ experiences and perceptions of primary care benzodiazepine prescribing and undertook a ‘meta-synthesis’ of these studies. ‘Meta-synthesis’ is “a family of methodological approaches to developing new knowledge based on rigorous analysis of existing qualitative research findings,” [17,18] used to synthesise and build upon these [16,19]. We used a ‘thematic synthesis’ approach to meta-synthesis [20] (see ‘data synthesis’ section below) in order to improve our understanding, and to inform policy and practice relating to primary care benzodiazepine prescribing [19,21,22].

### Search strategy

We systematically searched seven databases for relevant papers: MEDLINE, CINAHL, Social Science Citation Index, Science Citation Index, PsycINFO, Sociological Abstracts and AMED. We adapted our search strategy for MEDLINE for the other databases (Table 1). All databases were searched by October 2011.

**Table 1:** Medline search strategy

**Line number**	**Search term**
1	Qualitative
2	Interview*
3	Focus group*
4	Theme*
5	Experience*
6	MH qualitative research
7	MH interviews as topic+
8	MH attitude of health personnel
9	Attitude*
10	S1 OR S2 OR S3 OR S4 OR S5 OR S6 OR S7 OR S8 OR S9
11	MH benzodiazepines+
12	Benzodiazepine*
13	MH anti-anxiety agents
14	MH hypnotics and sedatives
15	z-drug*
16	z drug*
17	BZD
18	Anti-anxiety agent*
19	Anti anxiety agent*
20	Antianxiety agent*
21	Non-benzodiazepine*
22	Nonbenzodiazepine*
23	Non benzodiazepine*
24	Temazepam
25	Nitrazepam
26	Lormetazepam
27	Zopiclone
28	Zaleplon
29	Zolpidem
30	Eszopiclone
31	S11 OR S12 OR S13 OR S14 OR S15 OR S16 OR S17 OR S18 OR S19 OR S20 OR S21 OR S22 OR S23 OR S24 OR S25 OR S26 OR S27 OR S28 OR S29 OR S30
32	MH general practice+
33	MH general practitioners
34	MH physicians, family
35	MH physicians, primary care
36	MH nurses+
37	General practi*
38	Family practi*
39	Family doctor*
40	Primary care
41	Nurse*
42	S32 OR S33 OR S34 OR S35 OR S36 OR S37 OR S38 OR S39 OR S40 OR S41
43	S10 AND S31 AND S42 + Limiters: 1990 – 2011

*a wildcard search for any words which begin with the letters that precede the asterisk.

+the search term has been `exploded' to include all references indexed to that term and narrower terms beneath it.

### Inclusion and exclusion criteria

Studies were included if: they involved qualitative analysis of GP or practice nurse experiences of prescribing benzodiazepines; published between January 1990 and August 2011 to ensure relevance to current practice and the historical context of benzodiazepine prescribing; published in a European language; conducted in Europe, the United States, Australia or New Zealand; and in a primary care setting. We excluded quantitative studies published outside these dates, languages and settings.

**Figure 1:**
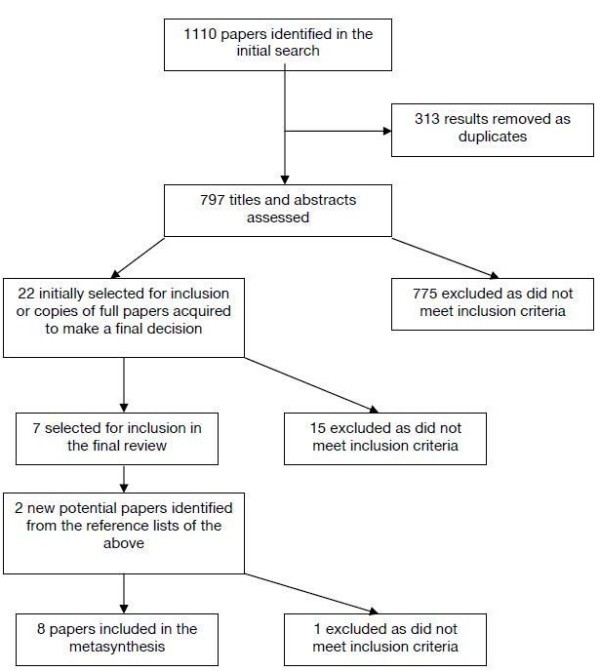
Study selection process.

We identified eight papers meeting these inclusion/exclusion criteria from 1110 potential papers after removing duplicates, examining the titles and abstracts (where available) or full papers (where relevance was unclear) and references of included papers (Figure 1).

### Data extraction and quality assessment

Two researcher pairs (SA and HC; CS and SYC) independently extracted data on study aims, setting, sample, theoretical perspective, data collection method and analysis. We appraised study quality using the Critical Appraisal Skills Programme (CASP) qualitative research checklist focusing on design, sampling, data collection, reflexivity, ethics, data analysis, findings and the value of the research [23]. Agreement on quality ratings was achieved through discussion to reach a consensus where there was initial disagreement on whether a particular criterion had been met.

### Data synthesis

‘Thematic synthesis’ was initially undertaken by the researcher pairs in three stages. Firstly we carried out line-by-line inspection of study ‘results’ to identify codes based on the meaning and content of each line. Second, we organised codes into a hierarchy of broader descriptive themes supported by NVivo 8 [20]. Finally, we developed analytic themes to build a model which sought to ‘go beyond’ the findings of the original papers to directly address the research question [20]. At each stage codes and themes were discussed to ensure consistency and agreement and the final synthesis was conducted by the wider multidisciplinary team with backgrounds in psychology, sociology and medicine.

## Results

### Data extraction

Eight studies were included from seven countries published between 1993 and 2010 (Table 2). These were based on GP data only, despite inclusion criteria also encompassing nurse prescribing. Seven studies involved semi-structured interviews and one used focus groups. One study [24] contained data from benzodiazepine users, and another included observational and quantitative measures [25] which were not included in the analysis.

**Table 2:** Description of studies included in the review

**Title and authors**	**Year of publication**	**Country**	**Aims**	**Sample**	**Method of data collection**	**Method of data analysis**	**Theoretical perspective**
**Anthierens et al.,** The lesser evil? Initiating a benzodiazepine prescription in general practice: a qualitative study on GPs’ perspectives	2007	Belgium	To investigate the views of GPs on why they initiate benzodiazepine prescriptions and their views about non-medical alternatives	35 GPs from a variety of practice settings (urban/rural)	5 focus groups	Focus groups were audio taped and transcribed verbatim. Analysed by 3 researchers using systematic content analysis. Themes were derived directly from the data rather than through an *a priori* framework. Also did deviant case analysis	Phenomenological. Researchers doing the analysis were from different disciplines - psychologist, sociologist and GP
**Cook et al.,** Physicians’ perspectives on prescribing benzodiazepines for older adults: a qualitative study	2007	USA	“To understand factors influencing chronic use of benzodiazepines in older adults” (p303)	33 primary care physicians in the Philadelphia area. Sought a range of levels of experience and practice settings	Semi-structured interviews	Interviews were audio taped and transcribed verbatim. Coded by a multidisciplinary team. 28 were face-to-face and 5 were telephone interviews. Used narrative analysis	Unclear
**Damestoy et al.,** Prescribing psychotropic medication for elderly patients: some physicians’ perspectives	1999	Canada	To explore “physicians’ perceptions and attitudes and the decision-making process associated with prescribing psychotropic medications for elderly patients” (p143)	9 physicians (from 12) who offered medical consultation in private apartment buildings for elderly people in a suburban region of Montreal	Semi-structured interviews	Interviews were taped and transcribed verbatim. Analysis continued until saturation was obtained for most categories. They refer to grounded theory, but there is insufficient information to confirm that they followed this perspective	Principals of Grounded Theory
**Dybwad et al., ** Why are some doctors high-prescribers of benzodiazepines and minor opiates? A qualitative study of GPs in Norway	1997	Norway	The authors state that they aimed “to form a basis for hypotheses and build theories about prescribing, in order to investigate how high-prescribing doctors can legitimize their own prescribing pattern” (p361).	18 high-prescribing GPs and 10 medium/low prescribers	Semi-structured interviews.	Interviews were audio taped and transcribed verbatim and analysed by the interviewer. Codes were derived from the data rather than a priori	Phenomenological
					GPs completed AUDIT and estimated their own prescribing volume. Observed in the practice each interview was conducted in and did a questionnaire for respondent characteristics		
**Parr et al.,** Views of general practitioners and benzodiazepine users on benzodiazepines: a qualitative analysis	2006	Australia	To gain a “more detailed understanding of perceptions relating to starting, continuing and stopping benzodiazepine use” (p1238) from the perspective of both users and GPs, and to compare the views of these groups	Convenience sample of 28 GPs and 23 benzodiazepine users from a range of locations in the tropical holiday and regional centre of Cairns	Semi-structured interviews	Interviews were audio taped and transcribed verbatim, and notes were taken during interviews. The notes were used for 4 GPs and 2 service users s due to equipment failure. Uses the Consensual Qualitative Research Approach. The article describes the different steps, but does not label them	Unclear
**Rogers et al.,** Prescribing benzodiazepines in general practice: A new view of an old problem	2007	UK	To give a brief history of the controversy surrounding benzodiazepine prescribing. To report a qualitative study of recent GP views on the use of benzodiazepines. To discuss the outcomes of this study in relation to “the general context of psychotropic drug responses to the psychosocial features of mental health problems’” (p182)	Purposive sample of 22 GPs, 15 male and 7 female GPs - newly qualified practitioners and GPs who had been practicing for some time and from a variety of practice settings across one English city. Captured a range of different ages, but was a bias towards younger GPs	Semi-structured interviews	Interviews were taped and transcribed. Themes derived through discussion by 5 researchers, with themes being modified by reading and re-reading transcripts	Unclear
**Smith,** General medical practitioners and community pharmacists in London: Views on the pharmacist’s role and responsibilities relating to benzodiazepines	1993	UK	To investigate GP's perceptions of the roles and responsibilities of community pharmacists in relation to promoting sensible use of benzodiazepines	Random sample of 22 GPs in London selected from lists held by he Family Health Service Authorities (out of 35 asked to participate)	Semi-structured interviews	Interviews were taped and transcribed. Data were collected and analysed using a coding frame.	Unclear
**Subelj et al.,** Prescription of benzodiazepines in Slovenian family medicine: a qualitative study	2010	Slovenia	To investigate how high-prescribing family physicians explain or justify their prescribing of benzodiazepines	Random sample of 5 family physicians with volumes of prescriptions larger than 4000 defined daily doses per month and 5 with volumes smaller than 2000 defined daily doses per month	Semi-structured interviews	Interviews were taped and transcribed verbatim. Very little information on how themes were then derived. There is a broad description on the methodology; they probably used thematic analysis but did not label it as such	Unclear

### Quality assessment

CASP criteria were independently rated ‘zero’ or ‘one’ by rater pairs. All papers scored 9 or 10 (out of 10) on CASP (Table 3). Cohen’s kappa 0.25 indicated initial discrepancy between pairs - this was resolved through discussion (as detailed above). No studies were excluded because all met the quality assessment.

**Table 3:** Quality assessment of papers

**Authors**	**UK CASP rating**	**Belgian CASP rating**	**Final joint CASP rating**
Anthierens et al., (2007)	9	10	10
Cook et al., (2007)	9	9	9
Damestoy et al., (1999)	9	9	8
Dybwad et al., (1996)	9	9	9
Parr et al., (2006)	10	10	10
Rogers et al., (2007)	9	10	9
Smith, (1993)	9	10	9
Subelj et al., (2010)	9	9	9

### Data synthesis

**Figure 2:**
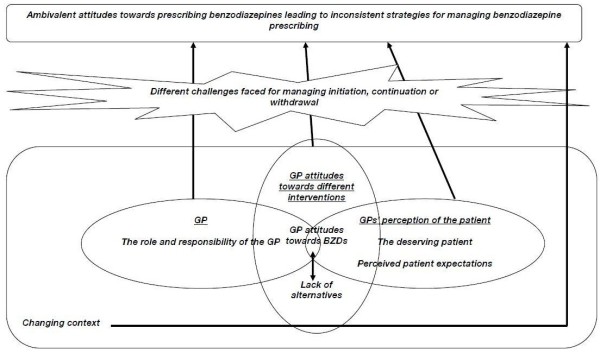
Explanatory model based on analytic themes.

We identified seven analytic themes namely: ‘the changing context of benzodiazepine prescribing’, ‘the role and responsibility of the GP’, ‘GP attitudes towards different interventions’, ‘the ‘deserving’ patient’, ‘perceived patient expectations’, ‘different challenges faced for managing initiation and withdrawal’, and ‘ambivalent attitudes towards prescribing benzodiazepines leading to inconsistent management strategies for prescribing benzodiazepines’. As shown by the arrows and the overlap between the shapes in this figure, there was some interaction between themes. The latter theme was shaped by a number of processes underlying prescribing practice which are identified in the remaining themes and the model (Figure 2) described in detail below.

### The changing context of benzodiazepine prescribing

GPs perceived the context for benzodiazepine prescribing decisions had changed over time because of changing: norms of practice, evidence, guidance (national and local), introduction of new drugs (e.g. selective serotonin reuptake inhibitors) and services, legal regulatory frameworks and the societal attitudes for treatment of conditions including anxiety, depression and insomnia. This changing context, which (as shown by the overlap in shapes in Figure 2) underlies many of the other themes identified, included two key aspects. Firstly, GPs stated being better informed about risks of benzodiazepines - a historical culture of prescribing that was optimistic to benefits and naïve about risks had largely been replaced by an attitude of scepticism:

“The respondents considered that this early optimistic therapeutic ethos has now been replaced with one of cautiousness as indicated by this respondent:

“I think there’s been an interesting change in the last 20 years in that I think you could say that there was a certain naivety and that if you, if anybody, now that I’m 50 said to me, ‘Here’s a great drug for anxiety which people can take long term and doesn’t have any addictive effects’ I simply wouldn’t believe them. But the fact was that when I qualified that was what we believed” [26].

Secondly, GPs now treated more patients, including those with mental health problems, previously treated in secondary care:

“While recently psychiatrists predominantly have focused on the management of psychosis, at the time of the emergence of the benzodiazepine problem they would regularly see outpatients with problems of anxiety and depression who were not, as now, managed in primary care” [26].

The changing context had influenced how GPs perceived their role and responsibilities towards their patients.

### Role and responsibility of the GP

Some GPs felt a sense of responsibility for past (which they now considered) poor prescribing practices, whilst others thought that this negative perception was overstated. There was tension between wanting to help patients, and feeling responsible for minimising BZD use:

“GPs…regarded BZD-prescribing as one of the most demanding and uncomfortable tasks in their clinical work. The discomfort was stated as a feeling of doing something almost illicit with prescribing because of the restrictive attitudes of both society and of the health authorities” [25].

“I think in some ways it has gone too far the other way in that GPs these days can be too afraid to use [a benzodiazepine]. I mean if the worst thing you can say about these drugs is that they have a potential for dependency, I don’t think that is a good enough reason to withdraw” [26].

GPs varied in the extent to which they accepted responsibility for past prescribing and/or wished to modify their practice in light of increasing evidence against long-term benzodiazepine use; some blamed others for inappropriate prescribing they had inherited:

“The origin of the problems of over-prescribing and turning a blind eye to the addictive features of the drugs were not always accepted as having been the sole responsibility of GPs; psychiatrists were seen as the source of the prescription habit. The respondents were keen to emphasize that a focus on GPs has been unreasonable, given that the psychiatric profession was deemed to be responsible for initiating and legitimizing the use of the drugs” [26].

GPs’ desire to support distressed patients was expressed as empathy through giving a prescription:

“If somebody comes along and says, ‘Here’s a good drug’ and the patients like it, then people are tempted to use it because it treats our own pain as well as our patients’ pain, ‘cos we want to help people and make people feel better. So if we give people something and make them feel better, then everybody seems to be happier” [26].

GPs felt responsible for prescribing appropriately but varied in attitudes to (correcting) ‘past’ prescriptions. Individual GPs often had explicit ‘rules’ about conditions for which they were willing to prescribe benzodiazepines, illustrated in the following two patient-related themes.

### The ‘deserving’ patient

GPs often managed the tension between minimising prescribing and their responsibility to help patients on a case-by-case basis. They needed to justify giving or withholding benzodiazepines, expressed in the literature through the concept of the ‘deserving patient’. In one study [6] participants were reluctant to indicate under what circumstances they would prescribe; but elsewhere, GPs described characteristics of those who might legitimately use benzodiazepines:

“GPs…reported that the majority [were] elderly and female, and many were continuous users with long histories of treatment by other doctors. Many of the continuous users suffered from multiple diseases, including both somatic and psychiatric disorders, and often a complexity of psychosocial problems. Patient[s] (sic) with anxiety and insomnia were prevalent [25].

‘Deserving patients’ included those who might elicit public sympathy whereas:

“Undeserving groups were those who do not elicit sympathy in the public eye and implicated GPs felt moral and legal responsibility. GPs labelled drug addicts and alcoholics as ‘undeserving’ patients. Substance abuse was a key clinical feature to attend to in decision making” [26].

This translated into different approaches for elderly compared with younger patients:

“A number of physicians appeared to have different rules and strategies for prescribing these medications in older versus younger adults, and were more tolerant of long-term use in the elderly” [6].

GPs often felt greater (or perceived greater public) sympathy but also felt a lack of alternatives for older compared with younger patients:

“Physicians thought their older adult patients would resist or be unable to pursue mental health referrals for multiple reasons ranging from stigma to financial and transportation difficulties” [6].

GPs varied in their estimation of the balance of adverse drug effects, including risk of addiction/abuse, against potential benefits for older patients:

“In the end, physicians believed that the advantages of continuing benzodiazepines in the elderly outweighed the problems” [6].

“Low-prescribers were more aware of cognitive impairment in the elderly and likelihood of falls and consecutive hip fractures” [27].

‘Deserving’ patients were also defined in terms of conditions such as bereavement or incurable or complex problems:

“For continuous prescribing of benzodiazepines and minor opiates, cure should have been abandoned. The patient is labelled as ‘beyond cure’ and can be placed in a ‘side track’, a label that permits palliative treatment” [25].

### Perceived patient expectations

Prescribing was influenced by how doctors *perceived* patients’ expectations, motivation and ability to cope. Expectations were sometimes assumed rather than directly discussed:

“Physicians anticipated resistance in response to even broaching the topic of taper/discontinuation with an older patient. Prospects ranged from questioning the doctor’s authority and competence, to minimization of potential negative side effects, to finding another doctor who was willing to prescribe it” [6].

Some patients were felt to be better able or motivated to cope without benzodiazepines or engage with alternative treatments than others:

“…these types of people and they tend not to want to help themselves, you know they won’t take hypnotherapy and they won’t go to yoga classes and they won’t do anything else. They just want a quick fix” [24].

A GP knowing a patient well and/or empathising with their situation, increased the likelihood of breaking previous ‘rules’ about what constituted a ‘deserving patient’:

“The following case shows that in some contexts the normal aversion to offering the drugs to patients with a drink problem noted earlier can be overridden by some GPs sympathetic to the personal plight of some patients: *The commonest is people with those long-term life problems, bad unhappy marriages, maybe have a partner who’s a drinker, has a drinking problem themselves. It’s hard to generalize but, yeah, you know, domestic violence, dysfunctional relationships, whatever. People end up on them and stay on them*” [26].

### GP attitudes towards different interventions

The treatment choices that GPs made in response to their perceptions of their patients, their patients’ expectations, and their own role and responsibilities were also influenced by their own attitudes and beliefs about different interventions. This ranged from disbelief that benzodiazepines would solve the patient’s problem to seeing drugs as the ‘lesser evil’ – particularly for psycho-social problems:

“A complex psychosocial situation is often the cause of the distress and the GP feels powerless in such situations. But the resolution of these problems does not always belong within the medical sphere; nevertheless, GPs look for a medical solution and they find BZDs to be the “lesser evil”: *You have to think that if you were in their situation you would not know what to do either. In this situation this person needs a BZD to give him some support for the things that are unbearable”*[28].

Benzodiazepines were viewed as safe or unsafe and effective or ineffective depending on the professional or personal experience of the GP. Negative attitudes towards benzodiazepines were based on their perceived risks:

“The addiction is so well known about that I think we all would just try and avoid using them for that reason” [26].

Others viewed the potential for dependence or adverse effects as less of a problem:

“GPs stated that dependence was not really a problem for first-time users. BZDs were seen as an efficient, cheap, and easy option that does not have too many side-effects” [28].

The ‘fast acting’ and effective nature of benzodiazepines for some GPs made them preferable to other forms of treatment:

“Sometimes it’s the easiest choice for people to feel best quickly. They feel better fast” [6].

Attitudes were also affected by GPs’ personal use of benzodiazepines:

“Your own attitude towards and experiences of the product definitely has an effect on prescribing. We, ourselves, take a lot of benzodiazepines” [28].

GPs perceived a lack of alternative treatments, depending on their knowledge of alternatives and their views about validity or effectiveness of non-pharmacological options for particular patients:

“GPs also feel uncertain how to deal with psychosocial problems, as a result of insufficient training: *I have to do a lot of “psycho” Whether I want it or not but I haven’t got the training for it. What do I do? I prescribe…”*[28].

“Scepticism regarding nonpharmacological approaches to the treatment of conditions such as anxiety and insomnia was expressed by the physicians interviewed. They identified common mild alternatives (e.g. warm milk, not watching violent movies before bed) and considered them to be ineffective for elderly people with chronic problems and thought that psychotherapeutic approaches were “doomed to failure”. Thus, the decision to prescribe medication was often seen as the most effective way to help the patient” [29].

In many cases, the alternatives used were ‘pills’ as these were thought to have benefits including placebo effects:

“GPs looked mainly for alternatives within their medical sphere. A wide range of medication such as antidepressants or neuroleptics was seen as an alternative. Other GPs were more inclined to use plant extracts because of a lower risk of dependence but at the same time they acknowledged a placebo effect” [28].

Some alternatives were seen as less ‘valid’ due to inaccessibility, stigma and costs for patients:

“Issues identified by less than five GPs included the need for non-stigmatised services; difficulty referring to other services, especially where there were stringent admission criteria; absence of feedback from other agencies; limited access to alternate services in rural and remote areas and a lack of time or resources to provide counselling, especially due to the absence of remuneration for doing so” [24].

The greater time GPs required to address patients’ (psychosocial) issues through alternative treatments (and the lack of remuneration for alternative treatments) compared with GPs’ view of benzodiazepines as a ‘fast acting’ solution for both the patient and themselves was another barrier:

“Physicians cited time constraints as both promoting benzodiazepine use and impeding discontinuation efforts. *A benzodiazepine becomes a quick fix because you don’t have time, this is what they want, they don’t feel good, here it is. It numbs them up and you’re not gonna get a phone call afterwards, you’re not gonna get anything, you’ll see them in a month, here’s your renewal, see ya later*” [6].

### Different challenges for managing initiation and withdrawal

GPs’ attitudes towards benzodiazepine prescribing, and behaviour, were moderated by the different challenges of a new prescription, continuing previous treatment or withdrawing drugs. The context of GPs’ practice, their view of their role, the perceived risks and effectiveness of benzodiazepines or alternative treatments, and the patient all influenced whether or not a GP chose to initiate, continue or withdraw benzodiazepines. Particular patient attributes, including old age, multiple conditions, and being perceived as a ‘deserving patient’ also increased the pressure to prescribe and gave a rationale to do so:

“being well acquainted with the patient further reduces the possibility to discontinue the drug, since they can understand their need for BZD support in particular situations” [27].

Time limited consultations (a barrier to alternative interventions), or the prospect of unhappy patients leaving for other practices, influenced whether or not GPs initiated withdrawal:

“She’s been on it for years, I’ll just give her what she asks for and I won’t have to sit here and explain things for twenty minutes about why I want to get her off. ‘Cause it is, it is an effort and time and frustration trying to get people off of these things. So maybe it’s just the path of least resistance” [6].

Not all GPs were concerned about the potential loss of patients:

“I’m not afraid of losing patients. I write a prescription only if it’s necessary” [27].

Withdrawal was problematic as GPs felt compelled to provide an alternative to benzodiazepines as well as applying a withdrawal strategy – something many had experienced failure with:

“Lacking strategies for both successful taper and alternative treatment, physicians did not want to withhold a medication that provided ongoing relief to the patient” [6].

### Ambivalent attitudes towards prescribing benzodiazepines leading to inconsistent strategies for managing prescribing

Overall, GPs were ambivalent towards prescribing benzodiazepines because of the issues described above, ranging from those who rarely prescribed, to those who did not see a problem with prescribing benzodiazepines. For most GPs, located in the middle of this continuum, these were complex decisions leading to conflicting pressures about whether or not to prescribe:

“These descriptions were discussed in relation to two imperatives in tension with one another: the moral obligation to ensure a programme of humane withdrawal; and the strict need to restrict access to a wider population. This tension is managed within the daily working constraints of GPs” [26].

These pressures led GPs to adopt a variety of management strategies from minimising benzodiazepine use to using tacit or explicit rules (heuristics) to justify prescribing. Complexity and conflict resulted in inconsistency between GPs in the strategies employed and how these were applied. Many GPs stated that they attempted to prescribe small quantities of benzodiazepines for short periods particularly when issuing new prescriptions, advising patients of the risk of adverse effects:

…I actually tell people when I am giving them a short course say for a crisis, that I only want you to take these for 4 or 5 days and then throw the rest of them away because they are habit forming” [24].

Some GPs saw it as part of their role to wean patients off drugs:

“A legitimate and expected role of the GP’s role now is to wean people off drugs to which they had inadvertently become addicted, for whatever reason, in the past” [26].

GPs’ tacit and explicit rules about benzodiazepine use included the following example:

“We do not prescribe BZD any more for people who have constant anxieties. We prescribe BZD in acute situations and always within a time limit” [28].

However these self-imposed rules were often inconsistently applied because of conflicting pressures, resulting in differences in behaviour between different GPs and within individual GPs’ practice.

## Discussion

### Main findings

Thematic synthesis enabled us to combine qualitative studies from different times and locations in a meaningful way to produce a model which clarifies our understanding of the complexities, challenges and potential solutions to primary care benzodiazepine prescribing.

The included papers originated from seven different countries, with different healthcare systems, but despite this, themes identified were broadly consistent across them. GPs were ambivalent towards prescribing benzodiazepines and inconsistently applied strategies for managing their use. Not only did different GPs vary in the extent to which they were willing to prescribe benzodiazepines, but *individual* GPs’ approaches varied. Making decisions on whether or not to prescribe was often uncomfortable, demanding and complex within the time and pressure constraints of daily practice.

GPs perceived a continually changing work context in which they were increasingly aware of the risks of benzodiazepines but more often encountered patients, perhaps previously managed in secondary care, who might require them. GPs felt a desire and responsibility to help their patients and decisions about what form this should take (i.e. a prescription or a non-pharmacological alternative) was based on competing and sometimes contradictory factors.

Concepts such as patient-centred practice and perceptions of patients’ expectations of benzodiazepine prescribing competed with GPs rationing role, autonomy, attitude to benzodiazepines, and wish to maintain good doctor-patient relationships – sometimes through giving patients their desired ‘quick-fix’ [30,31]. GPs faced different challenges during initiation, continuation or withdrawal of these drugs (Figure 2).

### Strengths and weaknesses

This is the first meta-synthesis of qualitative studies of benzodiazepine prescribing. Qualitative research encompasses a broad range of methods and philosophical positions, and our included studies were heterogeneous in terms of participants, geographical setting and time. Consequently, it may be problematic to generalise the findings and the particularities of a study may be lost in a meta-synthesis.

We have addressed these potential criticisms by including descriptions of each study (Table 2) and exploring the current relevance of the ideas gleaned (see below). We also employed techniques used for enhancing the validity of primary research studies, for example, using multiple investigators and considering the potential biases that their background might introduce in the analysis. Moreover, we provided direct quotations from included papers to ground our interpretation in the original studies [32] and have considered ‘negative cases’ within each of our themes. Using this transparent approach we were able to reach deeper insights into influences on prescribing and how this might be improved than could be gained from reading any of the included papers in isolation.

### Findings in relation to other studies

In his critical incident study of uncomfortable prescribing decisions, Bradley stated, “any attempt to influence the prescribing behaviour of doctors ought to be based on a thorough understanding of how prescribing decisions are actually made” [33]. It was the combination of factors influencing decisions whether or not to prescribe, rather than any single factor, which made it difficult [33]. This study similarly highlights tensions in the processes underlying decisions about benzodiazepine prescribing explaining why it proves such a difficult task.

GPs often made prescribing decisions in the context of uncertainty and in the short timescales and pressures of the consultation. Consequently they sometimes generalised from their experience of prescribing and withdrawing patients from benzodiazepines, to assume what patients’ views of risks and benefits would be - an example of Kahneman’s ‘representativeness’ heuristic [34]. Similarly, GPs justified giving a prescription to what has been described in previous literature as ‘deserving’ patients [26,35]: this may be an example of an ‘affect’ heuristic – where the difficult question of ‘should I prescribe’ has been replaced with an easier one of ‘is this a deserving patient’.

Research on the effect of empathy on decisions made for others shows that greater empathy with others leads to more impulsive decisions made for them [36]. Thus, whereas doctors may, in general, be more conservative in their decisions for patients than they would be for themselves, strong feelings of empathy for the patient may lead doctors to be more likely to offer a prescription.

GPs may also make *assumptions* about patients’ expectations rather than eliciting them directly, a notion which has been described previously in relation to antibiotics [37] and hypnotics [38]. Their attitudes about benzodiazepines and perceptions of the balance between the ease of prescribing and the risks and benefits may vary with each patient. Moreover, even when a GP does not believe that benzodiazepines will be an effective solution, they may still decide to prescribe due to a belief that there is a lack of valid alternatives for that patient, or a fear that any attempt to change a patient’s (perceived) preference of drugs over alternatives would be time-consuming or lead to the patient seeking another doctor.

The use of heuristics together with contextual limitations in terms of short consultation times, remuneration for treatment in some countries et cetera may lead to long-term prescribing contrary to clinical guidelines.

### Implications for future practice and research

In building our model of processes underlying current prescribing practices, we sought to identify ways to improve adherence to current clinical guidance which recommends use of psychological treatments for insomnia first-line and short-term use of benzodiazepines only. This section details our recommendations in relation to the role that GPs, education/training providers, and service providers can play in improving adherence to current clinical guidance.

Firstly, we ask what GPs can do to improve their adherence to current prescribing guidelines given the tensions and contextual limitations described above. The metasynthesis has shown that doctors sometimes erroneously assumed that patients wanted drug treatment and/or would be resistant to withdrawal whereas some patients preferred not to use or wished to discontinue drugs [8,38]. Such patients wanted to be heard, taken seriously and given explanations of treatments [38,39]. One way of addressing this issue would be if GPs are made more aware, through both education and continuing self-reflection, of the impact of empathy and perceived patient expectations on their decision-making; and encouraged to directly explore patients’ ideas and expectations and discuss treatment options, explaining their benefits and drawbacks [38]. Despite constraints of short appointment times, this approach may lead to reduced workload long-term with fewer patients returning for repeat prescriptions. This potential benefit is something which could be the subject of future research.

GPs also expressed concerns about the accessibility of some alternatives to benzodiazepines for some patients. The legitimacy of this concern could be considered in future research working with patients to seek feedback on their perceptions of the accessibility of alternative treatments and with other professionals, such as mental health workers and pharmacists [40], to provide such alternatives.

Secondly, we ask what educational providers can do to improve GPs’ adherence to current prescribing guidelines. The papers report mixed findings in terms of GPs’ willingness to engage in further training, particularly due to the time constraints that they face [27,29]. However, we suggest that there would be a benefit to addressing GPs’ knowledge deficits through increasing education and training around current guidelines, the adverse effects of benzodiazepine use, and alternative forms of treatment. Such training should be targeted at high benzodiazepine prescribers in particular and should include information on the evidence for non-pharmacological alternatives such as face-to-face or self-help (including computerised) cognitive behavioural therapy for insomnia (CBT-I). In addition, it should raise GPs’ awareness of the impact of empathy and perceived patient expectations on decision-making as outlined above.

Finally, we feel that there is a clear role for service providers in improving adherence to current prescribing guidelines in the long-term by increasing the availability and accessibility of face-to-face and computerised CBT-I.

Further primary research may be needed to confirm these findings, and a fuller understanding of the dynamics of benzodiazepine prescribing decisions should also consider patients’ perceptions.

## Conclusion

Benzodiazepine prescribing decisions in primary care are complex, demanding and uncomfortable. This study has increased our understanding of why this is so and has the potential to inform future interventions to improve adherence to prescribing guidance and improve prescribing of benzodiazepines through enhanced education and training of professionals on benzodiazepine use and withdrawal, greater provision of alternatives to drugs, reflective practice, and better communication with patients. Finally, our findings have wider implications for difficult prescribing decisions since similar issues often apply to other prescribing contexts.

## Competing interests

The authors declare that they have no competing interests.

## Authors’ contributions

All the authors (CS, SA, HC, SYC, TC, ANS) participated in conception and design of the study. ANS was the principal investigator. CS, SA, HC and SYC undertook the systematic review, identified the studies, undertook the quality assessment and extracted data for the metasynthesis. All authors were involved in the analysis and interpretation of the data. CS wrote the first draft of the paper. All the authors contributed to drafts, critically revised the manuscript and approved the final version.

## Authors’ information

ANS and TC are academic general practitioners. HC and SYC are psychologists. SA and CS are social scientists.

## Pre-publication history

The pre-publication history for this paper can be accessed here:

http://www.biomedcentral.com/1471-2296/14/191/prepub
